# Hybrid sequential treatment of a giant serous mesenteric cyst: description of a case and review of the literature

**DOI:** 10.1093/jscr/rjac397

**Published:** 2022-09-09

**Authors:** Giorgio Lucandri, Giulia Fiori, Sara Lucchese, Flaminia Genualdo, Vito Pende, Massimo Farina, Paolo Mazzocchi, Emanuele Santoro

**Affiliations:** Department of Surgery, San Giovanni-Addolorata Hospital, Rome, Italy; Department of Surgery, San Giovanni-Addolorata Hospital, Rome, Italy; Department of Surgery, San Giovanni-Addolorata Hospital, Rome, Italy; Department of Surgery, San Giovanni-Addolorata Hospital, Rome, Italy; Department of Surgery, San Giovanni-Addolorata Hospital, Rome, Italy; Department of Surgery, San Giovanni-Addolorata Hospital, Rome, Italy; Department of Surgery, San Giovanni-Addolorata Hospital, Rome, Italy; Department of Surgery, San Giovanni-Addolorata Hospital, Rome, Italy

**Keywords:** simple mesenteric cyst, benign mesenteric cyst, abdominal pain, palpable abdominal mass, simple mesothelial cyst

## Abstract

Mesenteric cysts are uncommon benign abdominal tumors that may extend from the root of the mesenteric layers of the gastrointestinal tract into the retroperitoneum or the peritoneal cavity; they are usually asymptomatic and often represent an occasional finding. Definitive diagnosis is confirmed by the surgical intraoperative view and by histopathological examination. Surgical excision of the cyst is the treatment of choice. We present a case of a female patient who presented with back pain and a palpable abdominal mass. Due to large size of the mass and its contiguity with midline, patient underwent an hybrid combined surgical technique, with a first open phase followed by a laparoscopic excision. Complete surgical removal of the cyst was successfully performed without bowel resection, intraoperative spillage of cystic content and without morbidity. Histopathology confirmed diagnosis of simple mesenteric cyst. We strongly recommend a combined approach whenever a large intraperitoneal benign cystic lesion has been diagnosed.

## INTRODUCTION

Mesenteric cysts are very uncommon and literature usually deals with single case report or little consecutive series. An incidence of 1/140.000 in surgical departments and 1/20.000 in pediatric departments has been reported [1]. They usually originate from the mesenteric layers of the GI tract and arise from its root into the retroperitoneum and/or the peritoneal space; they may develop from small bowel mesentery (60%), colic mesentery (24%) or may affect entirely the retroperitoneum (15%) [2, 3]. Mesenteric cysts are often asymptomatic and are discovered during radiological tests performed for different reasons; common findings are their slow and progressive growth as well as absence of infiltrative attitude. They may present with abdominal distension, diffuse abdominal pain, symptoms and/or signs of compression of surrounding structures, such as subocclusion, hydronephrosis, nausea or dyspepsia [2]. In ~10% of patients, mesenteric cysts exhibit clinical signs of acute abdomen due to twisting of the cyst on its peduncle and impairment in vascular supply or to spontaneous or traumatic rupture of parietal wall [2, 4]. Mesenteric cysts may present as single or multiple, uni- or multi-locular in structure and may be classified as serous, chylous, hemorrhagic, lymphatic and infected because of different types of content; according to this subclassification, serous cysts are localized in the ileal or colic mesentery, chylous cysts mostly affect the jejunal mesentery, while post-traumatic hemorrhagic cysts can develop everywhere into the gut [4]. Neoplastic transformation occurs rarely (3%) [4]. Mesenteric cysts affect more frequently females in the fifth decade of life as in the patient we recently observed and successfully treated: her young age, remarkable size of the cyst at imaging and probable absence of malignancy led us to select a combined sequential open-laparoscopic surgical approach in which criteria of safety, surgical radicality and mini-invasivity could be satisfied.

## CASE PRESENTATION

**Figure 1:**
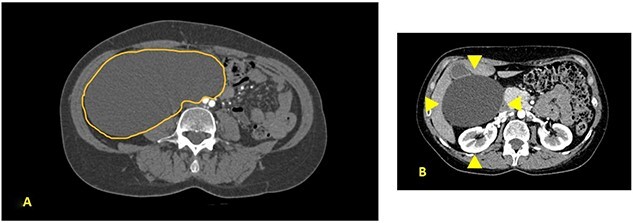
(**A**) Contrast CT scan; gross oval-shaped mass with clear fluid content (10–15 HU) and regular margins, measuring 15 × 10 cm in maximum diameters; (**B**) evidence of liver and gallbladder compression (arrowheads).

**Figure 2:**
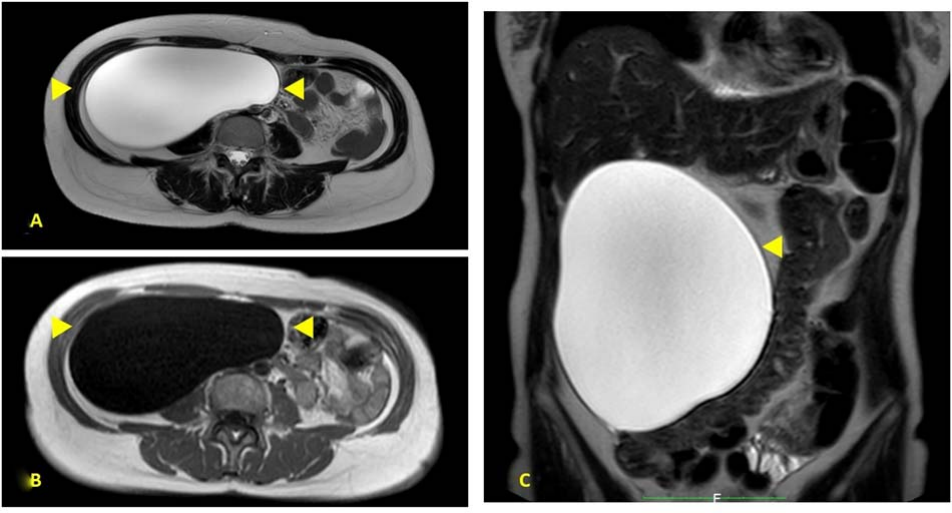
(**A**) Prohance® MR; main lesion appears hyperintense on T2-weighted sequences (arrowheads); (**B**) main lesion appears hypointense on T1-weighted sequences (arrowheads); (**C**) coronal T2-weighted sequences confirmed main radiological findings: size 15 × 20 cm, thin aspect of envelope, absence of infiltrative aspect and gross displacement of surrounding structures.

**Figure 3:**
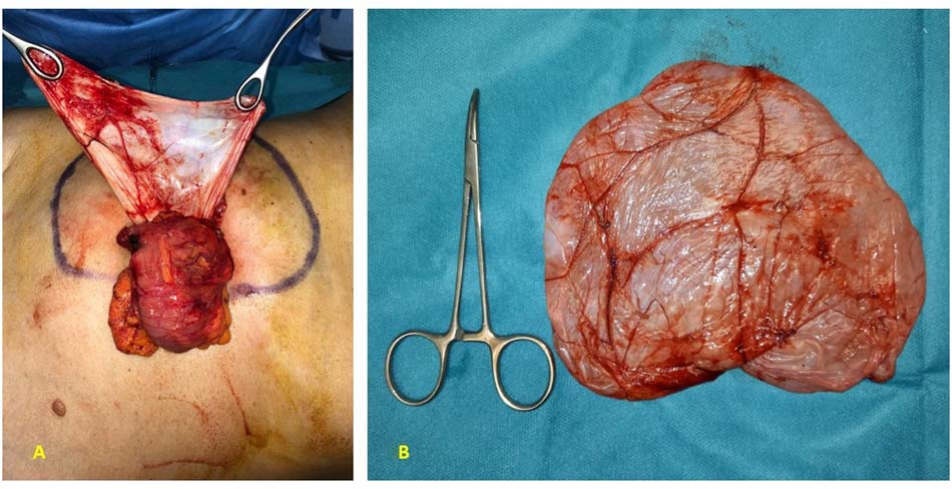
(**A**) At operative setting, cyst has been empty of its content and partially dissected from ascending colon; (**B**) macroscopical appearance of resected specimen: mesenteric cyst with thin and translucent envelope.

A 52-year-old female was visited at our outpatient service on November 2021 for increasing back pain (4.5 points in the Pain Scale Chart) and the presence of a huge palpable mass occupying the entire right abdomen; she complained of a decade history of recurrent abdominal pain and repeated ultrasound (US), revealing an increasing abdominal cystic-like bulk with fluid content (18 × 8.5 cm. at last US). Neither previous abdominal surgery nor significant familiarity was referred; canalization and food intake were described as regular. At physical examination, a large mass occupying right abdomen could be easily detected; basic laboratory panel and tumoral markers were within range, while body mass index and KPS were, respectively, 21.2 and 90%. Angio-computed tomography (CT) scan revealed a ‘Gross oval-shaped mass with clear fluid content (10–15 HU) and regular margins, measuring 15 × 10 cm. in maximum axial diameter and cranio-caudal extension of ~20 cm, surrounded by a thin hyperdense wall in the absence of intralesional enhancement on delayed phase’ (Fig. 1A). Several structures were displaced as an effect of tumoral enlargement (lower aspect of the right kidney, inferior vena cava, right psoas muscle, gallbladder, right ascending colon, some jejunal loops, head of the pancreas and common bile duct; Fig. 1B). The entire right abdomen resulted as occupied by this huge cystic lesion, which caudally reached the homolateral iliac space. At Prohance® MR, both T1- and T2-weighed sequences confirmed main radiological findings (size 15 × 20 cm, thin aspect of envelope, absence of infiltrative aspect and gross displacement of surrounding structures; Fig. 2A–C). On these bases, preoperative diagnosis of simple mesenteric cyst was hypothesized and the patient was addressed to surgical treatment. Hybrid open-laparoscopic approach was planned: peritoneal space was reached via a midline of 4-cm supraumbilical access and a wall protector-retractor device (Alexis®—Applied Medical) was positioned. Cystic wall lied just beneath surgical access: after a 2/0 Vicryl 2/0 purse string, cyst was incised and 1600 ml of clear-watery content was removed and sent for cytology. Cystic envelope was sharply dissected from ascending colon (Fig. 3A) and was reintroduced into the abdomen. After closing the Alexis® device, a 12-mmHg pneumoperitoneum was induced with the positioning of the 10-mm Alexis® trocar and 2 5-mm working cannulas. Deepest aspect of the cystic wall was completely dissected from the hepatic flexure of the colon and from the second duodenal portion by using a US device and the specimen was removed and sent for histological examination (Fig. 3B). Post-operative was uneventful and the patient was discharged on the second post-operative day. Cytology result was negative for malignancy; at histological examination, a thin-walled cystic formation, translucent at macroscopical appearance was described. Maximum diameter of the empty lesion was 15 cm. Definitive diagnosis was ‘simple serous cyst of the mesentery’ (IHC staining Calretinin−; CD10−).

## DISCUSSION

Clinical and radiological finding of a gross endoperitoneal cystic lesion is uncommon: differential diagnosis with pancreatic pseudocyst and cystic tumors, peritoneal hydatid cyst, pelvic malignancies and aortic aneurysm is mandatory [5]. US represents the first-level examination, showing cystic aspect of the mass, its main diameters, presence of intralesional septa and echogenicity of its content [6]. Angio-CT and MR provide further information on relationships with surrounding organs and main vessels, cystic content, aspect and thickness of the envelope, presence and distribution of septa and coexistence of a solid component [6]. The most appropriate treatment of large mesenteric cyst is surgical removal; alternative treatments, such as unroofing, marsupialization, drainage with or without sclerotherapy, endoscopic resection, enucleation and excision associated with intestinal resection, should be reserved for emergency settings for patients with anesthesiological contraindications to surgery and for situations of close contiguity with main retroperitoneal vessels [7–11]. In the case herein reported, large sizes of the lesion and its contiguity with midline led us to perform an hybrid-sequential approach: minilaparotomic and laparoscopic. Firstly, a mini access (unavoidable anyway for next removal of the specimen) was obtained: it allowed the removal of cyst content, reduced size of the cyst and minimized the risk of its rupture. An adequate working space was so obtained, allowing a safe laparoscopic dissection of the deepest aspect of the cyst from ascending colon and second duodenal portion, avoiding inappropriate spillage of cyst content. We strongly recommend such combined approach whenever a large intraperitoneal benign cystic lesion has been diagnosed.

## CONFLICT OF INTEREST STATEMENT

None declared.

## FUNDING

None.
